# Elevated Systemic Pentraxin-3 Is Associated With Complement Consumption in the Acute Phase of Thrombotic Microangiopathies

**DOI:** 10.3389/fimmu.2019.00240

**Published:** 2019-02-25

**Authors:** Eszter Trojnar, Mihály Józsi, Zsóka Szabó, Marienn Réti, Péter Farkas, Kata Kelen, George S. Reusz, Attila J. Szabó, Nóra Garam, Bálint Mikes, György Sinkovits, Blanka Mező, Dorottya Csuka, Zoltán Prohászka

**Affiliations:** ^1^Research Laboratory, MTA-SE Research Group of Immunology and Hematology, 3rd Department of Internal Medicine, Hungarian Academy of Sciences and Semmelweis University, Budapest, Hungary; ^2^Complement Research Group, Department of Immunology, ELTE Eötvös Loránd University, Budapest, Hungary; ^3^Department of Haematology and Stem Cell Transplantation, Central Hospital of Southern Pest, National Institute for Hematology and Infectious Diseases, Budapest, Hungary; ^4^1st Department of Pediatrics, Semmelweis University, Budapest, Hungary; ^5^MTA-SE Pediatric and Nephrology Research Group, Budapest, Hungary

**Keywords:** pentraxin-3, C-reactive protein, thrombotic microangiopathies, hemolytic uremic syndrome, thrombotic thrombocytopenic purpura, alternative pathway, complement consumption

## Abstract

Pentraxin-3 (PTX3) and C-reactive protein (CRP) have been shown to regulate complement activation *in vitro*, but their role has not been investigated in complement consumption *in vivo*. Thrombotic microangiopathies (TMA) are often accompanied by complement overactivation and consumption, therefore we analyzed the relation of the systemic pentraxin levels to the complement profile, laboratory parameters and clinical outcome of TMA patients. We determined the PTX3 and CRP levels, complement factor and activation product concentrations in blood samples of 171 subjects with the diagnosis of typical hemolytic uremic syndrome (STEC-HUS) (*N* = 34), atypical HUS (aHUS) (*N* = 44), secondary TMA (*N* = 63), thrombotic thrombocytopenic purpura (TTP) (*N* = 30) and 69 age-matched healthy individuals. Clinical data, blood count and chemistry were collected from medical records. To determine the *in vitro* effect of PTX3 on alternative pathway (AP) activation, sheep red blood cell-based hemolytic assay and AP activity ELISA were used. We found that PTX3 levels were elevated in the acute phase of STEC-HUS, aHUS and secondary TMA, whereas PTX3 elevation was exceptional is TTP. Conversely, a significantly higher median CRP was present in all patient groups compared to controls. PTX3, but not CRP was associated with signs of complement consumption *in vivo*, and PTX3 significantly decreased the AP hemolytic activity *in vitro*. Our results provide a detailed description of acute phase-TMA patients' complement profile linked to changes in the systemic pentraxin levels that may support further molecular studies on the function of PTX3 in disease pathogenesis and add to the laboratory assessment of complement consumption in TMA.

## Introduction

Pentraxin-3 (PTX3) and C-reactive protein (CRP) are fluid phase pattern recognition molecules that have been shown to interact with the complement system on multiple levels. PTX3 consists of a unique N-terminal domain (1) and a highly conserved C-terminal pentraxin-like domain that is shared with CRP and allows octamer formation of the secreted PTX3 monomers through inter-chain disulfide bonds (2). Prompt release of PTX3 from neutrophil granulocytes is mediated at local sites of activation (3), whereas its enduring production is regulated via gene expression induction in innate immune cells and endothelial cells (3). Native CRP, a member of the short-pentraxin protein family, is stored as a pentamer in the endoplasmic reticulum of resting hepatocytes (4). Upon inflammatory stimuli CRP is secreted into the circulation and phosphocholine binding on target cell membranes induces the disassembly of the pentameric structure to CRP monomers in a calcium-dependent fashion (4).

PTX3 may facilitate phagocytosis of pathogens and clearance of cellular debris through the activation of the classical (CP) and lectin pathways of complement (2) upon binding to surface-associated mannan-binding lectin (5), ficolins, collectins (6) and C1q (7). Conversely, its interaction with C1q in the fluid phase restricts unwanted complement activation (3, 7). PTX3 also may recruit functionally active complement regulatory proteins, such as factor H (FH) (8) and C4b-binding protein (C4BP) (9) to the surface of apoptotic cells, which in turn facilitates C3b or C4b degradation and phagocytosis. Hence, by FH binding, PTX3 may prevent alternative pathway (AP) amplification and activation of the terminal pathway on non-activator surfaces *in vitro* (1). However, *in vivo* disease models of infection and tissue injury reported contradictory observations on the role of PTX3 during the inflammatory response. Both endogenous and exogenous PTX3 were shown to attenuate leukocyte recruitment and decrease apoptosis in experimental models of kidney and myocardial tissue injury (10, 11), whereas excess PTX3 was shown to intensify the inflammatory response in disease models of intestinal ischemia (12, 13) and certain respiratory pathologies (14).

CRP also has the ability to activate the CP of complement. Pentameric CRP however, may only bind solid phase C1q when complexed to phosphocholine (15), with concomitant restrain of the terminal pathway (16). By contrast, monomeric CRP may induce excess CP activation both *in vitro* and *in vivo* (15, 16), but at the same time it also allows for CRP to interact with the complement regulators C4BP, FH, but also with properdin (15, 17, 18), thus regulating both the CP and AP.

Thrombotic microangiopathies (TMA) are life threatening conditions that involve acute thrombocytopenia, hemolysis and organ impairment. Endothelial damage and subsequent microvascular thrombosis are key pathogenic factors in all forms of this disease (19, 20), despite differences in the clinical course and management of TMAs with distinct etiologies. Microvascular thrombosis has been linked to excessive complement activation in all forms of TMA (21–23) together with neutrophil activation and neutrophil extracellular trap (NET) release (24–28), which may provide excess PTX3 at the site of tissue injury (29) and thus influence the local complement activity.

Albeit numerous investigations have characterized the interaction of pentraxins with complement factors *in vitro*, no study has been designed so far to explore changes in the systemic level of pentraxins in complement mediated diseases, such as TMAs. Therefore, we performed a case-control study to determine the systemic levels of PTX3 and CRP in patients at the acute phase and remission of TMA. We explored the association between TMA-related complement consumption and circulatory pentraxin levels *in vivo* as well as the direct effect of PTX3 on AP activation *in vitro*, to reveal the potential role of pentraxins in complement mediated tissue injury. We further analyzed the relationship between the systemic level of pentraxins and TMA etiology, the clinical outcome of patients and classical laboratory markers of TMA.

## Methods

### Patient Selection and Sample Collection

**Figure 1:**
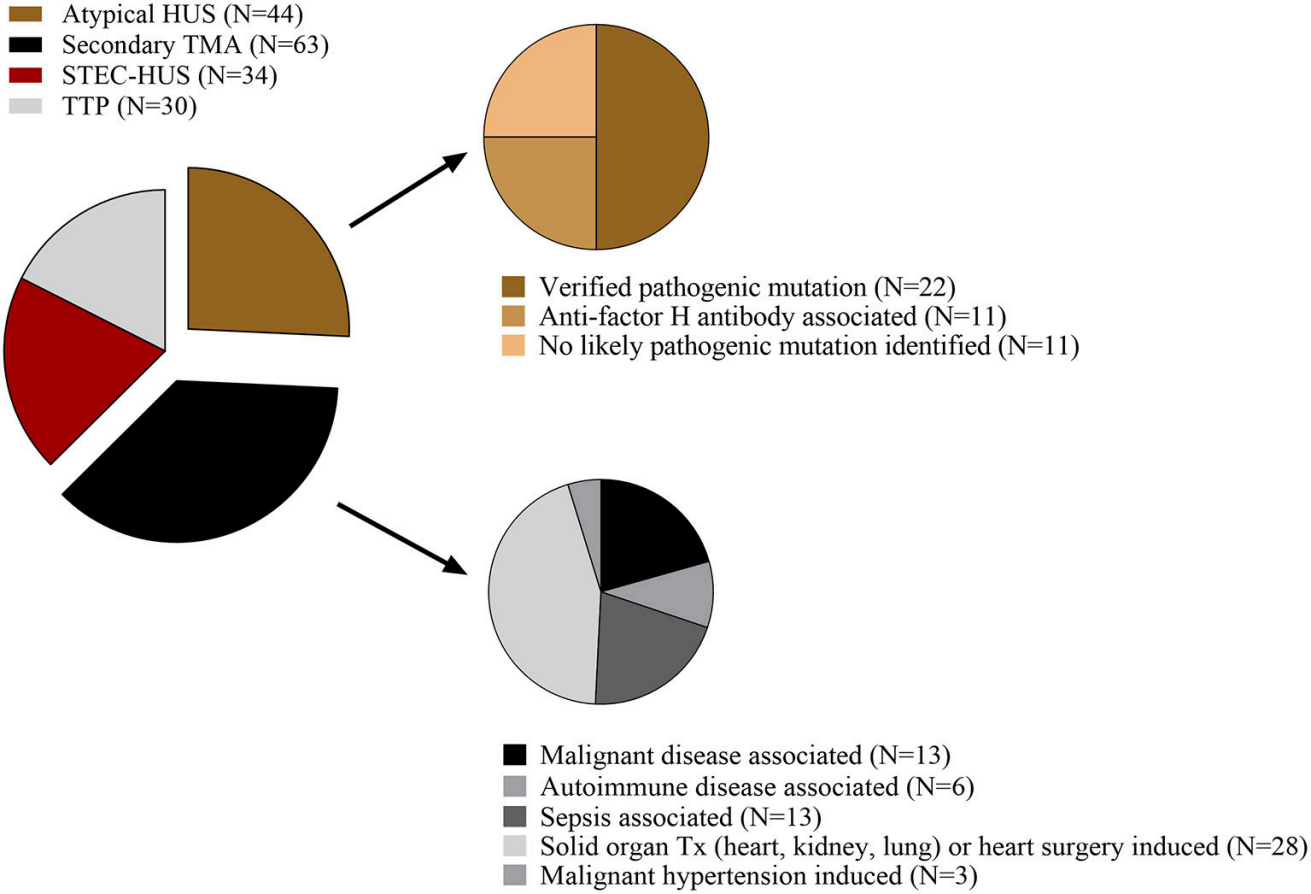
Representation of TMA disease etiology in the studied population. The number of participants per group (N) is shown as proportion of a whole. HUS, hemolytic uremic syndrome; STEC-HUS, Shiga-like toxin associated HUS; TMA, thrombotic microangiopathy; TTP, thrombotic thrombocytopenic purpura; Tx, transplantation.

171 TMA patients with acute disease flare were enrolled in this study. Serum and plasma samples from all subjects were collected prior to the start of plasma exchange therapy; however, in 16 cases fresh frozen plasma had been administered to the patients prior to sampling. For appropriate comparison, 69 age-matched healthy individuals were selected, none of whom showed clinical or laboratory signs of TMA or an acute phase reaction that could have influenced the measured laboratory parameters. Diagnosis of TMA was established based on laboratory signs of thrombocytopenia (<150 G/L), and microangiopathic hemolytic anemia. Patients were included in the study only if all of the above criteria were met. For stratification of patients by disease etiology the following groups were formed: STEC-HUS (*N* = 34), aHUS (*N* = 44), secondary TMA (*N* = 63) and TTP (*N* = 30) (Figure 1), based on additional diagnostic criteria detailed in the Supplementary Material. Exclusion criteria were ongoing plasma exchange or complement inhibitory therapy at the time of sample collection (during the first acute flare), or the lack of available blood sample. For additional details on the study population please see the methods section of the Supplementary Material. This study was carried out in conformity with the Helsinki Declaration. Written informed consent was obtained from all participants, and the study was approved by the Scientific and Research Ethics Committee of the Medical Research Council (ETT TUKEB) in Budapest, Hungary (8361-1/2011-EKU).

### Determination of Laboratory Parameters

Complement activity-, component-, regulator-, and activation product determinations, CRP and PTX3 measurements were performed in this study. The AP activity was determined with the commercially available WIESLAB Alternative pathway ELISA kit (EuroDiagnostica, Malmö, Sweden), while total complement classical pathway activity was assessed using the sheep-erythrocyte hemolytic titration test. C3, C4 and hsCRP were measured by turbidimetry (Beckman Coulter, Brea, CA), complement factors B, and I were determined by radial immunodiffusion assay. The level of the complement regulators C1q and FH and the titer of the anti-FH antibodies were measured using in-house ELISA techniques, described in detail elsewhere (22, 30, 31). A disintegrin and metalloproteinase with a thrombospondin type 1 motif member 13 (ADAMTS13) activity was evaluated by the application of the fluorogenic substrate FRETS-VWF73 (22). Commercially available kits were used to assess the levels of the complement activation products soluble C5b-9 (sC5b-9) and C3a (C3a des-arg) (Quidel, San Diego, CA) and for the measurement of PTX3 (R&D systems Minneapolis, MN). For the determination of CRP, PTX3, complement factor levels and pathway activities patient's sera were obtained. The complement activation products (sC5b-9 and C3a) were determined from EDTA anticoagulated plasma, whereas the ADAMTS13 activity was evaluated from sodium-citrate-anticoagulated plasma of the patients.

### *In vitro* Assessment of PTX3 Effect on AP Activation

We applied normal human serum (NHS) with additional recombinant human PTX3 in two established methods for the assessment of AP activity: the WIESLAB AP ELISA kit (EuroDiagnostica, Malmö, Sweden) and the C3 nephritic factor hemolytic assay (32), with modifications. The C3 nephritic factor assay was performed on washed sheep erythrocytes, where patient's samples were replaced by NHS spiked with recombinant human PTX3 (R&D systems Minneapolis, MN, USA) in gradually decreasing concentrations. Following a 20-min incubation of PTX3 with NHS, the solution was added to sheep erythrocytes. The formation of the C3 convertase was allowed within a 10-min incubation time at 30°C, and assembly of the terminal pathway membrane attack complex was achieved by the addition of undiluted rat serum to the cells, following multiple washes. After incubation at 37°C for 60 min, the extent of hemolysis was detected by reading the optical density (OD) at 412 nm. The effect of PTX3 on the assembly of C5b-9 on a plastic surface was assessed with the WIESLAB Alternative pathway ELISA kit (EuroDiagnostica, Malmö, Sweden). Similarly to the above, patient's sera were replaced by PTX3 spiked NHS, otherwise the assay was performed according to the manufacturer's instructions. To allow comparison of data, the hemolytic or AP activities in each experiment were expressed as ratio of the reference (mean OD of NHS with buffer control) in percentage.

### Statistical Analysis

Data analysis was performed using the GraphPad Prism version 6.00 (GraphPad Software, La Jolla, CA, www.graphpad.com). The statistical analysis applied for data comparison is indicated in each figure legend and detailed in the Supplementary Material.

## Results

### Patient Characteristics

This study was performed to determine the systemic level of CRP and PTX3 in 171 TMA patients in acute disease flare and to investigate the role of PTX3 in complement dysregulation *in vivo* compared to 69 age and sex-matched healthy individuals. Basic clinical and laboratory characteristics of the patients and controls are summarized in Table 1.

**Table 1:** Characteristics of the TMA patients and healthy controls.

**Characteristics analyzed**	**TMA**	**Healthy controls**	**Result of statistical comparison**
Number of individuals enrolled	171	69	NA
Age	35.2 (7.7–56.9)	33.0 (18.7–41.0)	*P =* 0.608
Sex (male/female in %)	43/57	48/52	NA
First acute episode (%)	93.6	NA	NA
31-days mortality (%)	11.7	0	NA
Complement C3 < 0.9 g/L (%)	49.7	0	NA
Complement FH < 250 mg/L (%)	25.7	0	NA
**LABORATORY PARAMETERS INDICATIVE OF ONGOING TMA**			
Red blood cell count (10^9^/L)	2.9 (2.6–3.4)	4.9 (4.6–5.2)	*p <* 0.001
Hemoglobin (g/L)	85 (75–97)	141 (134–152)	*p <* 0.001
Platelet count (10^9^/L)	46 (22–75)	262 (235–309)	*p <* 0.001
Lactate dehydrogenase (U/L)	1,819 (893–3,051)	Not done	NA
Creatinine (μmol/L)	188 (86–320)	71 (64–78)	*p <* 0.001
Carbamide (mmol/L)	16.9 (10.9–25.9)	4.5 (3.8–5.6)	*p <* 0.001
**PENTRAXIN LEVELS AND WHITE BLOOD CELL PROFILE**			
PTX3 level (μg/L)	5.19 (2.08–13.17)	1.08 (0.75–1.66)	*p <* 0.001
CRP level (mg/L)	16.9 (4.3–72.0)	1.4 (0.8–2.0)	*p <* 0.001
White blood cell count (G/L)	10.4 (7.1–15.3)	6.5 (5.4–7.9)	*p <* 0.001
Absolute neutrophil count (G/L)	7.1 (4.8–12.4)	4.0 (3.0–4.7)	*p <* 0.001
Absolute lymphocyte count (G/L)	1.4 (0.8–2.6)	2.0 (1.8–2.4)	*p <* 0.001
**COMPLEMENT PARAMETERS**			
ADAMTS13 activity (%) (Reference range: 67–147%)	38 (17–54)	Not done	NA
Classical pathway activity (CH50/ml)	57 (45–71)	70 (62–77)	*p <* 0.001
Alternative pathway activity (%)	86 (56–101)	101 (78–117)	*p <* 0.001
C3 level (g/L)	0.90 (0.68–1.15)	1.26 (1.18–1.47)	*p <* 0.001
C4 level (g/L)	0.23 (0.14–0.32)	0.34 (0.27–0.40)	*p <* 0.001
Factor H level (mg/L)	390 (245–513)	560 (462–692)	*p <* 0.001
Factor I level (%)	98 (81–123)	102 (92–108)	*p =* 0.192
Factor B level (%)	98 (73–116)	101 (91–113)	*p =* 0.791
C1q level (mg/L)	87 (56–112)	100 (80–124)	*p =* 0.020
sC5b-9 level (ng/mL) (Reference range: 110–252 ng/mL)	352 (265–517)	Not done	NA
C3a level (ng/mL) (Reference range: 70–270 ng/mL)	171 (120–259)	Not done	NA

*Characteristics and laboratory data of the TMA patients and healthy controls. Data are shown as median with interquartile range, results of statistical comparison are indicated with the respective p-value of the Mann-Whitney test. ADAMTS13, a disintegrin and metalloproteinase with a thrombospondin type 1 motif, member 13; CRP, C-reactive protein; FH, factor H; NA, not applicable; PTX3, pentraxin-3; sC5b-9, soluble C5b-9; TMA, thrombotic microangiopathy*.

Our study group consisted of TMA patients with the following etiologies: STEC-HUS (*N* = 34), aHUS (*N* = 44), secondary TMA (*N* = 63), and TTP (*N* = 30) (Figure 1). Over 90% of the admitted patients presented with the first acute episode of the disease. Blood samples were obtained from all patients preceding the start of plasma exchange or complement inhibitory therapy, although 16 patients received fresh frozen plasma prior to sampling. Figure 1 shows the distribution of patients with various etiologies in the aHUS and secondary TMA groups.

All acute phase-TMA patients presented with laboratory signs of hemolysis and thrombocytopenia (<150 G/L), with the lowest median platelet count (i.e., 16 G/L) in the TTP subgroup. ADAMTS13 activity was decreased in 79% of the patients and ADAMTS13 deficiency was present in all of the TTP patients. Organ involvement manifested in clinical and laboratory signs of kidney damage or neurological symptoms as a sign of central nervous system involvement in most of the TMA patients. Classical laboratory parameters indicative of ongoing TMA in each of the study groups are summarized in Supplementary Table 1.

### Pentraxin Levels in Acute Phase-TMA and Their Relation to the Laboratory Markers of Disease and Clinical Characteristics of Patients

**Figure 2:**
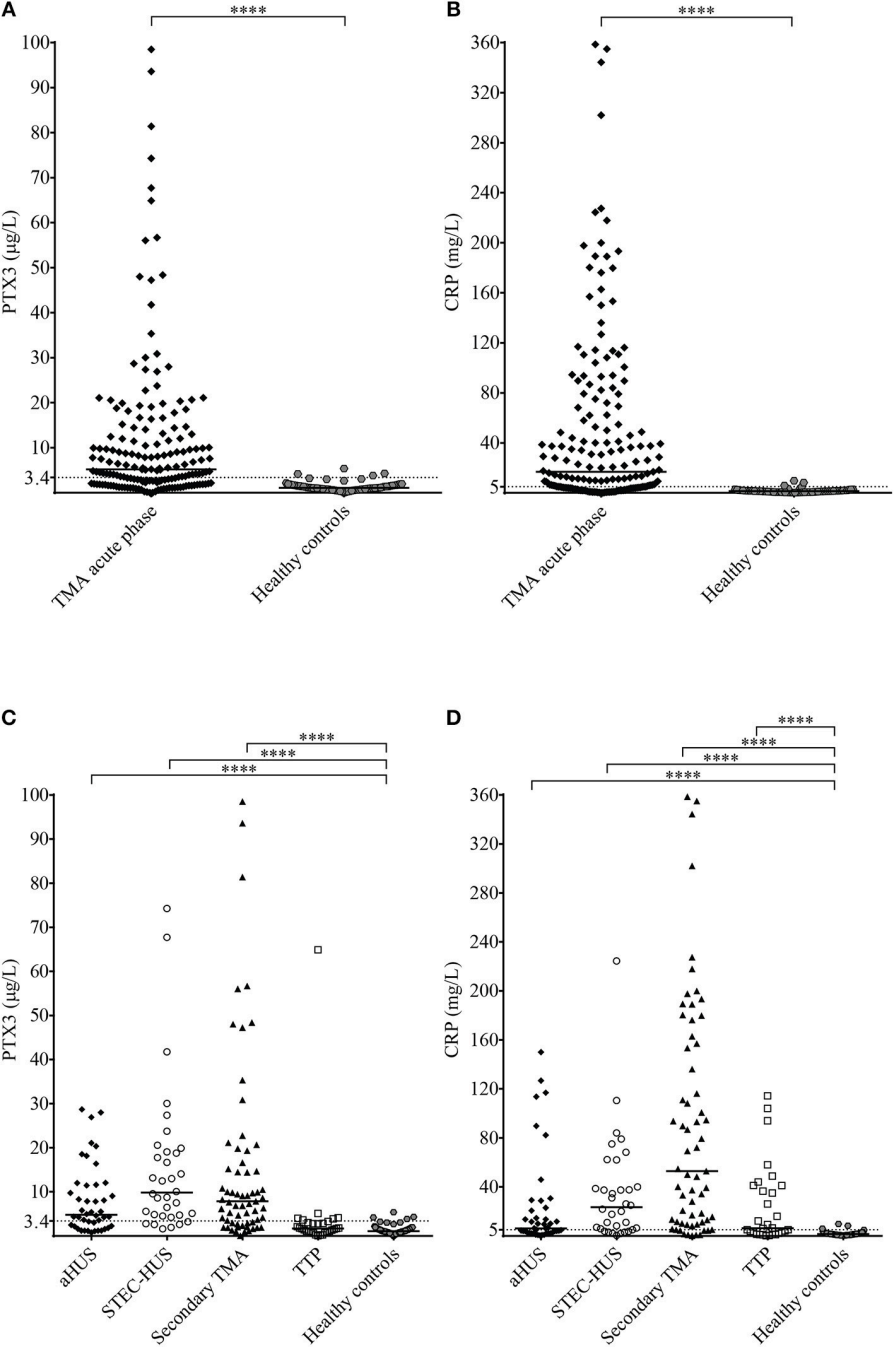
PTX3 and CRP levels in acute TMA vs. healthy controls. **(A,B)** PTX3 and CRP levels of TMA patients at the acute disease onset compared to pentraxin levels of healthy individuals. **(C,D)** PTX3 and CRP levels of acute phase-TMA patients grouped by disease etiology. Data are expressed as mean of technical duplicates, the horizontal line indicates the median of each group, while an intermittent line shows the calculated cutoff of each pentraxin, respectively. Statistical analysis was performed with the Mann Whitney test **(A,B)** or the Kruskal-Wallis test corrected for multiple comparisons using the Dunn's *post hoc* test **(C,D)**, respectively. Statistical significance is indicated by asterisks (****p < 0.0001). aHUS, atypical hemolytic uremic syndrome; CRP, C-reactive protein; PTX3, pentraxin-3; STEC-HUS, Shiga-like toxin associated HUS; TMA, thrombotic microangiopathy; TTP, thrombotic thrombocytopenic purpura.

We measured a significantly elevated median PTX3 level in acute phase-TMA compared to healthy controls (Figure 2A), with an elevated systemic PTX3 level in 64% of the acute phase TMA patients. CRP levels were also higher, exceeding the upper limit of normal range (5 mg/L) in 70% of TMA patients (Figure 2B). The calculated cutoff of CRP levels (5.01 mg/L) was equivalent to the upper limit of normal range (5 mg/L) used in our laboratory in frames of diagnostics, whereas the cutoff of PTX3 levels was determined based on the levels measured in the healthy control group and set as 3.40 μg/L (mean + 2 times the standard deviation of healthy controls).

**Figure 3:**
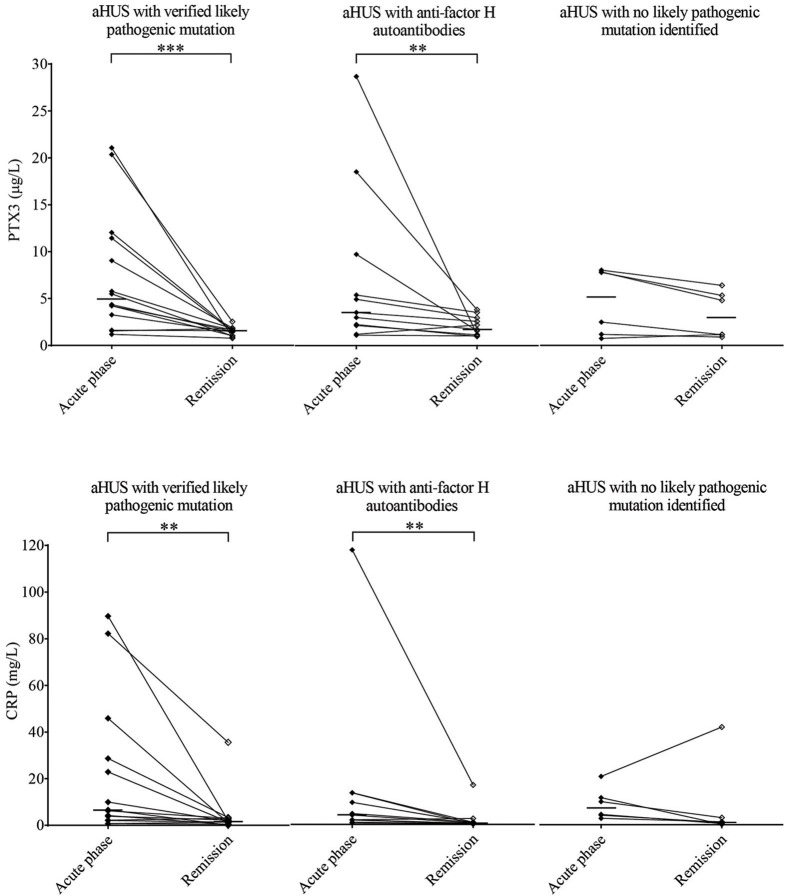
PTX3 and CRP levels in aHUS acute phase and remission. PTX3 **(A)** and CRP **(B)** levels of aHUS patients are shown in the acute phase (black squares) and in remission (empty squares) with a continuous line connecting the respective sample pairs, while the medians of each group are indicated by a horizontal line. Half of the patients (*N* = 22, left) had a confirmed likely pathogenic mutation, 11 patients presented with anti-factor H antibodies (middle) and by the rest (*N* = 11, right) no likely pathogenic mutation has been identified in the complement genes (*CFH, CFHR5, CFI, CD46, C3, CFB*), *THBD* or *DGKE*. Data points represent mean of technical duplicates, statistical analysis was performed with the Wilcoxon-signed rank test, statistical significance is indicated by asterisks (**p < 0.01; ***p < 0.001). aHUS, atypical hemolytic uremic syndrome; CRP, C-reactive protein; PTX3, pentraxin-3.

Elevated PTX3 and CRP levels could be detected in all etiology groups of TMA, although PTX3 elevation was exceptional in TTP, despite the elevated CRP level in 53% of the patients of this subgroup (Figures 2C,D). With further subdivision of the study groups, we found that the elevation of both pentraxins was independent of the molecular background in aHUS, since in each of the distinct aHUS subgroups PTX3 or CRP levels were significantly elevated compared to healthy controls (all *p* < 0.05, Mann-Whitney test) (Figures 3A,B), and we detected similar pentraxin levels in secondary TMAs with distinct etiologies, too (data not shown). PTX3 levels were associated with markers of disease activity and organ damage in TMA. We observed a positive correlation between lactate dehydrogenase and PTX3 levels, and a weaker yet significant correlation of the platelet count and laboratory signs of kidney damage to PTX3. By contrast, association between CRP and disease activity was not present, except a significant positive correlation to creatinine levels (Table 2). The parameters presented in Table 2 were entered into two multiple regression models to explore relationship between them and PTX3 or CRP, respectively. LDH (standardized regression coefficient beta = 0.299) turned out to be significant predictor of PTX3 in the multivariable model, whereas platelet and kidney function measures did not. For CRP, significant predictors were hemoglobin (beta = 0.183), platelet number (beta = −0.179) and creatinine (beta = 0.338) levels. Since platelet count is a reliable marker of disease activity in TMA, we further explored its relationship to the systemic pentraxin levels by grouped analysis of patients according to platelet counts at the time of admission. We found that irrespective of the classification, median PTX3 and CRP levels of all subgroups remained significantly elevated compared to healthy controls (Supplementary Figure 1). Furthermore, PTX3 and CRP showed a strong positive correlation to each other and to markers of systemic inflammation such as the white blood cell count and absolute neutrophil count of the patients (Supplementary Figures 2, 3). In the 16 FFP-treated patients enrolled in this study, administration of FFP did not results in an improvement of the clinical status or the classical laboratory signs of TMA until the time point of blood sample collection. We performed all our analysis with the exclusion of the FFP-treated patients as well, and it did not change any of our conclusions on the correlations observed at the acute phase of TMA.

**Table 2:** Correlation of the systemic pentraxin levels to laboratory markers of TMA.

**Laboratory parameters analyzed**	**PTX3**			**CRP**		
	**Spearman r**	***p*-value**	***N***	**Spearman r**	***p*-value**	***N***
Red blood cell count	0.08785	0.3380	121	0.02669	0.7714	121
Hemoglobin	0.007054	0.9329	145	0.07903	0.3447	145
Platelet count	**0.1975**	**0.0161**	**148**	−0.1107	0.1806	148
Lactate dehydrogenase	**0.299**	**0.0004**	**134**	0.003968	0.9637	134
Creatinine	**0.2421**	**0.0023**	**156**	**0.2266**	**0.0045**	**156**
Carbamide	**0.2011**	**0.0257**	**123**	0.1377	0.1289	123

*Spearman correlation analysis of PTX3 and CRP levels to laboratory markers of hemolysis, thrombocytopenia and kidney damage, characteristic to acute phase-TMA. Statistical analysis was performed by the Spearman correlation test, obtained r and p-values are indicated by each parameter, with the number of pairs analyzed. Significant correlations are highlighted in bold. Please note that blood count and chemistry data were not accessible by all the enrolled patients, therefore the correlation analyses might include less than the total number of patients (N = 171) in this study, CRP. C-reactive protein; PTX3, pentraxin-3; TMA, thrombotic microangiopathy*.

### Elevated Pentraxin Levels Normalize in Disease Remission

We obtained follow-up samples from 31 aHUS patients and 19 of the TTP patients. In over 80% of aHUS both PTX3 and CRP levels decreased in remission compared to the paired acute phase samples, but the extent of decline did not reach statistical significance in patients with no clarified molecular background of the disease (Figures 3A,B). The median PTX3 level also remained significantly higher in aHUS remission compared to the control group, while the CRP levels in remission were similar to that of healthy controls (Supplementary Figure 4). The initially low PTX3 levels of TTP patients showed no remarkable difference in remission, and the CRP levels also normalized in over 80% of the cases (Supplementary Figure 5).

### Association of the Median PTX3 Level With the Acute Phase Mortality

**Figure 4:**
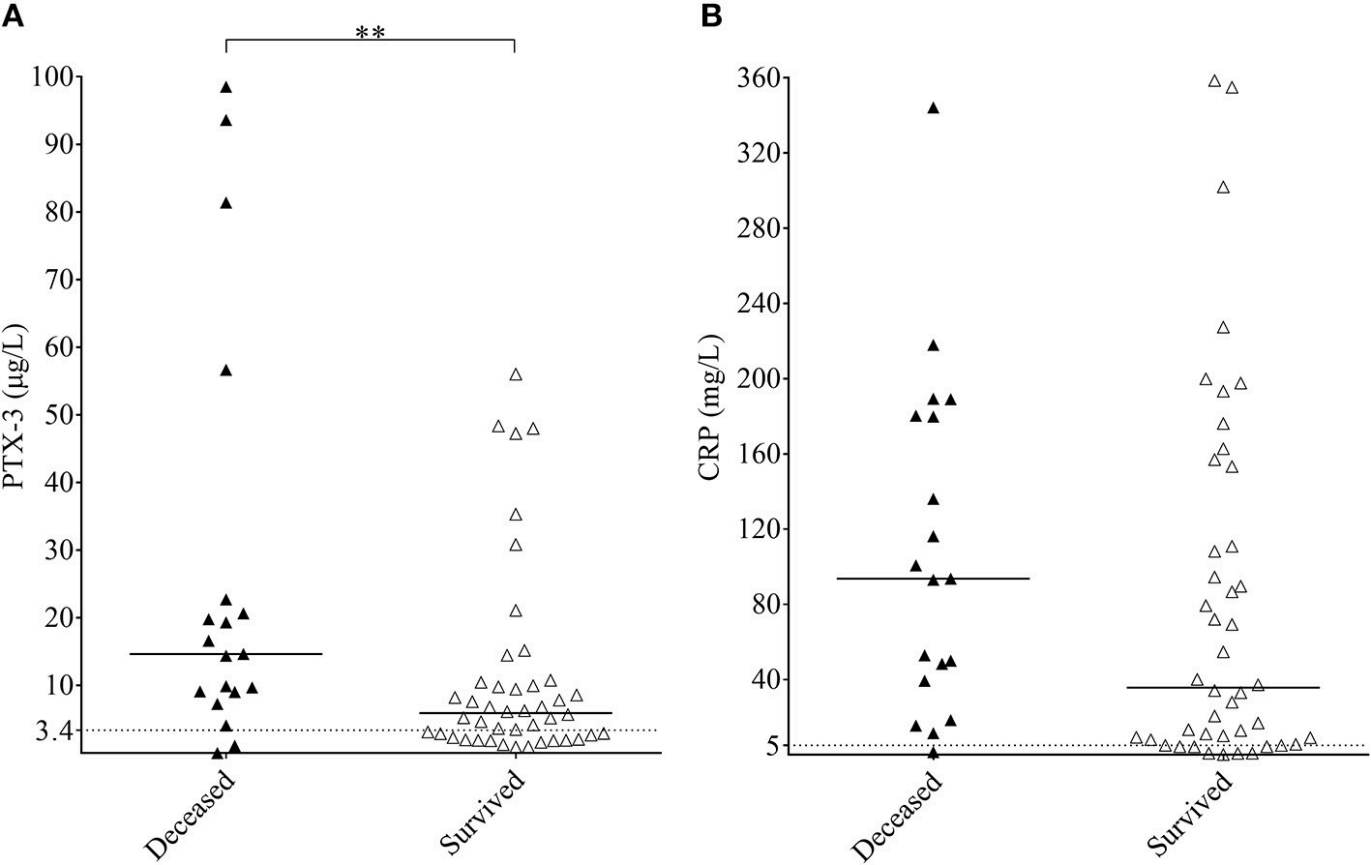
Association of the systemic pentraxin levels to acute phase mortality in secondary TMA. PTX3 **(A)** and CRP **(B)** levels of acute phase secondary TMA patients are shown, grouped based on the 31-day survival of patients (deceased, black triangles; survived, white triangles). Data are expressed as mean of technical duplicates, the horizontal line indicates the median of each group, while an intermittent line shows the calculated cutoff of each pentraxin, respectively. Statistical analysis was performed with the Mann-Whitney test. Statistical significance is indicated by asterisks (**p < 0.01). CRP, C-reactive protein; PTX3, pentraxin-3; TMA, thrombotic microangiopathy.

The overall 11.7% acute phase mortality arose from the high mortality rate of the secondary TMA group, which exceeded 30% within a 31-days period. No deaths occurred in the STEC-HUS or aHUS study groups and one patient died in the TTP group. The median CRP levels did not differ significantly in secondary TMA patients who survived the acute phase compared to those who did not, but the median PTX3 level was significantly higher in the deceased individuals compared to those who survived the first month of the TMA episode (Figures 4A,B). The optimum PTX3 cut-point was 9 μg/mL to differentiate patients who died during follow up, from those who survived [odd's ratio 3.08 (95% CI 1.02–9.33)]. One-by-one adjustment for key activity indicators showed that high PTX3 levels are hemoglobin and creatinine independent predictors of mortality, whereas dependent on platelet and LDH levels.

### Signs of Complement Consumption in Acute Phase-TMA and Their Association With the Systemic Pentraxin Levels

**Figure 5:**
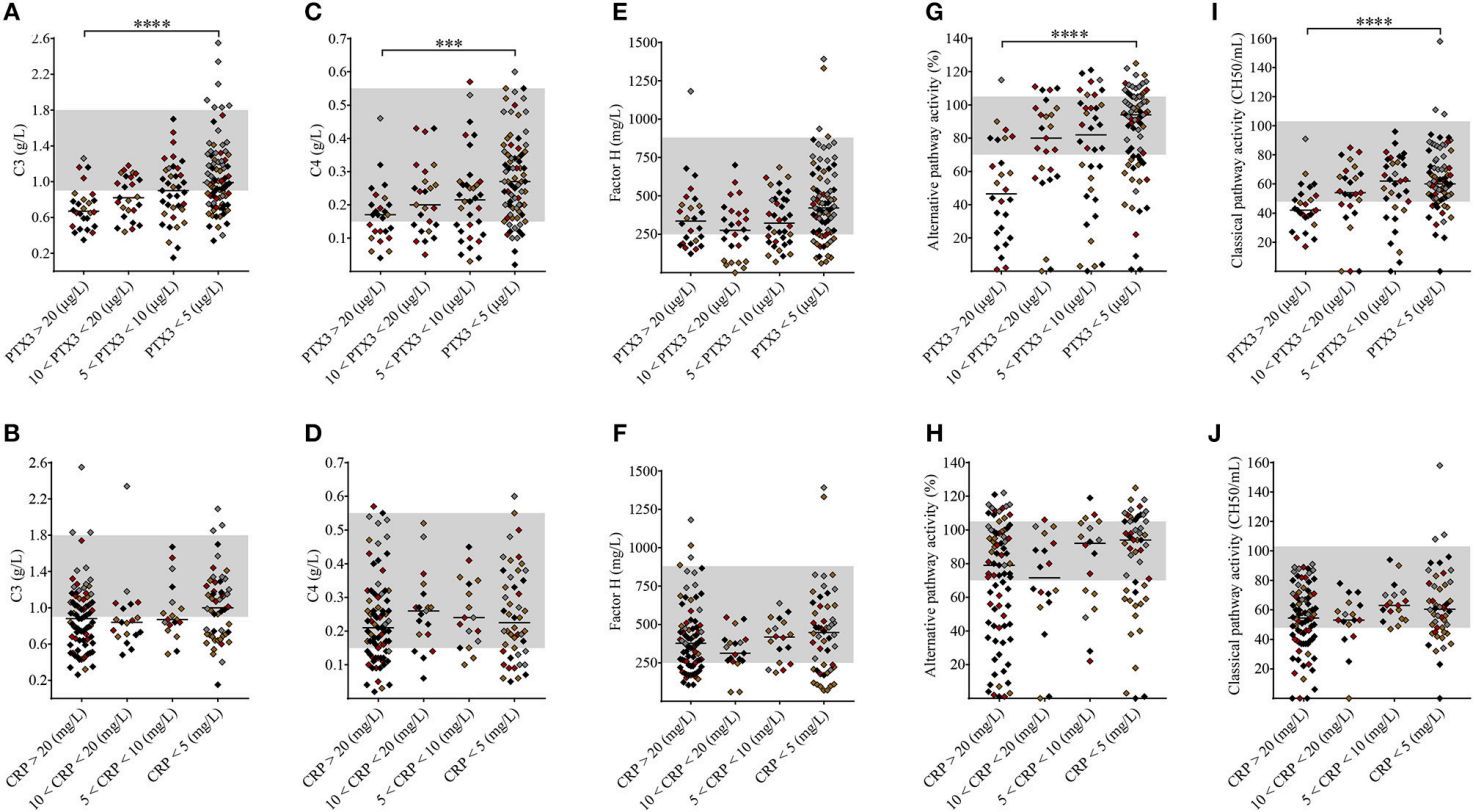
Association of the systemic pentraxin levels with laboratory signs of complement consumption. The degree of complement activation and consumption was assessed from complement factor levels (**A–F**: C3, C4, FH) and complement activity parameters **(G–J)** in TMA patients subdivided based on the measured systemic PTX3 or CRP levels, respectively. Data are expressed as mean of technical duplicates, the horizontal lines show the median of each group and the laboratory normal range is indicated with gray shading. The color of each data point indicates the specific form of TMA corresponding to Figure 1 (brown, aHUS; red, STEC-HUS; black, secondary TMA; gray, TTP). Statistical analysis was performed with the Kruskal-Wallis test corrected for multiple comparisons using the Dunn's *post hoc* test. ANOVA ***p* < 0.0001**, *p* = 0.1285, ***p* = 0.0002**, *p* = 0.6713, ***p* = 0.0358**, *p* **=** 0.2173, ***p* < 0.0001**, *p* = 0.0717, ***p* = 0.0001**, *p* = 0.053 for **(A–J)**, statistical significance of the Dunn's tests are indicated by asterisks (***p < 0.001, ****p < 0.0001) on the respective figure panels **(A–J)**. aHUS, atypical hemolytic uremic syndrome; CRP, C-reactive protein; PTX3, pentraxin-3; STEC-HUS, Shiga-like toxin associated HUS; TMA, thrombotic microangiopathy; TTP, thrombotic thrombocytopenic purpura.

Nearly 50% of the TMA patients presented with decreased C3 levels indicative of complement consumption (Table 1), while only 9% of the patients (15/171) showed no signs of complement alteration (with C3, C4, FH, C1q, factor I and factor B levels, CP and AP activities, and complement activation product levels within the laboratory normal range). To assess whether elevated pentraxin levels were associated with complement consumption in the acute phase of TMA, we grouped the patients based on PTX3 and CRP levels and observed a strong linkage between the gradual increase in PTX3 and signs of complement AP and CP consumption (Figure 5 and Supplementary Table 2). As a result of complement overactivation and complement factor consumption, both C3 and C4 levels were significantly lower in patients with PTX3 above 20 μg/L compared to those below 5 μg/L. If the relationships between decreased C3 and C4 levels to the elevated PTX3 (Figures 5A,C) were further analyzed in subgroups of patients stratified according to the most important confounder, i.e., LDH level (Supplementary Figure 6), similar associations were observed. Although the gradual increase of PTX3 was not accompanied by a decrease in the FH levels, complement CP and AP activities were significantly lower in patients with PTX3 above 20 μg/L compared to those with PTX3 below 5 μg/L. Moreover, patients with a PTX3 level exceeding 20 μg/L had a median AP and CP activity below the normal range indicating explicit complement consumption. By contrast, CRP levels did not show an association with any of the measured complement activity parameters.

### Influence of PTX3 on AP Activation *in vitro*

**Figure 6:**
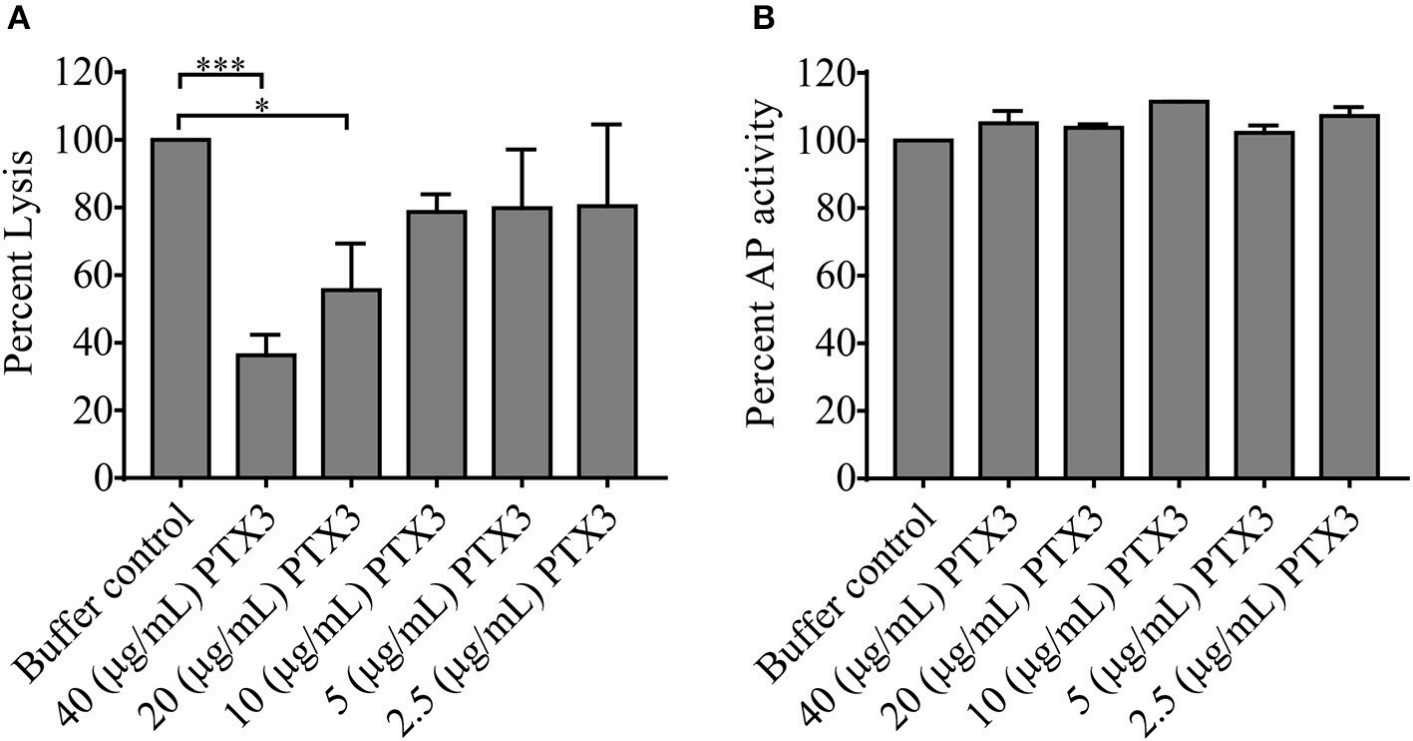
Effect of PTX3 on AP activity *in vitro*. The effect of recombinant human PTX3 on AP hemolytic activity **(A)** and AP-mediated C9 deposition **(B)** is shown in percent compared to buffer control added to pooled serum of healthy individuals. Data represent mean of 3 times repeated experiments with technical duplicates, error bars indicate the standard error of mean. Statistical analysis was performed with the Kruskal-Wallis test corrected for multiple comparisons using the Dunn's *post hoc* test. Statistical significance is indicated by asterisks (*p < 0.05; ***p < 0.001). AP, alternative pathway; PTX3, pentraxin-3.

*In vivo* complement consumption was accompanied by a gradual increase in the systemic PTX3 level in our acute phase-TMA patients. To explore the functional relevance of this phenomenon we tested whether PTX3 attenuates or stimulates the AP activity on the cellular surface. In a modified hemolytic assay (used to determine the C3 nephritic factor level) we built up the AP convertase on sheep erythrocytes and determined the hemolytic activity of NHS with the addition of recombinant human PTX3 or buffer control, respectively (Figure 6A). We found that addition of PTX3 significantly decreased the activity of the AP C3-convertase on sheep red blood cells. Conversely, addition of PTX3 to NHS did not influence AP activity on the surface of ELISA plates. Hence, no remarkable change was detected in C9 deposition through lipopolysaccharide (LPS)-induced activation of the AP (Figure 6B), whereas PTX3 alone did not bind to LPS in the ELISA wells (data not shown).

## Discussion

Our study investigated the role of PTX3 and CRP in association with complement consumption in the acute phase of TMA. We provide a detailed description of acute phase-TMA patients' complement profile linked to changes in the systemic pentraxin levels. We report that PTX3 elevation is present in the acute phase of STEC-HUS, aHUS and secondary TMA but is exceptional in TTP. Conversely, an elevation in the systemic CRP level is present regardless of disease etiology in the acute phase of TMA (Figure 2). Disease remission in aHUS was accompanied by a decline in the level of both pentraxins (Figure 3). However, while CRP decreased to values observed in healthy individuals, the median PTX3 level remained significantly higher in aHUS compared to controls (Supplementary Figure 4). In the remission of TTP no notable alteration of the PTX3 levels could be recorded (Supplementary Figure 5). We observed the highest acute phase mortality in secondary TMA patients, which was associated with high PTX3 but not CRP levels (Figure 4). TMA was accompanied by laboratory signs of complement activation and consumption in the majority of our patients. We show for the first time that AP and CP consumption is associated with elevated PTX3 in the acute phase of TMA (Figure 5). To explore a potential mechanism in the background of this observation, we confirmed *in vitro* that PTX3 limits AP activity on the surface of red blood cells, with no effect on terminal pathway assembly during LPS-induced AP activation on ELISA plates (Figure 6).

Microthrombus formation in TMA results in extensive inflammation that involves turnover of the complement and coagulation cascades together with the activation of innate immunity (21). The observed elevation of both pentraxins in acute phase-TMA and their strong positive correlation to the white blood cell and absolute neutrophil counts suggests that PTX3 and CRP production is induced in frames of the ongoing inflammatory response (Supplementary Figures 2, 3). TMAs have recently been linked to neutrophil cell activation and NET formation (24–28), as a component of which PTX3 may be released on demand from leukocyte infiltrates that accumulate at the site of endothelial damage (3, 29). Furthermore, local levels of PTX3 may increase via its production by injured endothelial cells (3), hence providing a possible dual-source of PTX3 during the acute phase of TMA. Conversely, increased CRP production may be attributed to the induction of a systemic inflammatory response that induces the release of acute phase proteins.

In approximately 60% of aHUS cases mutations to the complement genes or antibodies directed against the complement regulator FH account for the pathophysiological process leading to AP dysregulation and consumption (33, 34), whereas in the remaining one-third of the cases the molecular background remains unrevealed. Our aHUS cohort had a somewhat higher representation of autoimmune aHUS (25 vs. 10%) and a relatively small proportion of unexplained cases (25 vs. 30–40%) compared to the previously reported prevalence (33, 34). We had a notable number of patients with low FH level in our patient cohort. This arose from FH mutations and antibodies in aHUS, albeit patients with a low FH level were also present in STEC-HUS, secondary TMA and TTP, indicating the presence of complement dysregulation in multiple forms of TMA. Nonetheless, elevated pentraxin levels were present in all aHUS subgroups independent of the molecular etiology. PTX3 and CRP elevation was also prominent in STEC-HUS and secondary TMA, regardless of the heterogenic etiological background of the patients. However, PTX3 elevation was exceptional in TTP, albeit neutrophil cell activation together with complement dysregulation have been described in TTP (22, 28). Laboratory signs of kidney damage were also absent in 70% of the TTP patients, while most patients with other forms of TMA presented with a varying degree of kidney injury. Both acute and chronic kidney damage have been linked to the elevation of PTX3 (35), the lack of which in TTP could provide a possible explanation for the absence of PTX3 elevation in TTP. However, it cannot be excluded that additional factors arising from the distinct pathogenesis of TTP (34) have also contributed to the observed difference.

Secondary TMA patients in our study cohort had an overall 30.2% in-hospital mortality, which is comparable to observations reported in literature (23, 36). Acute phase disease mortality was associated with a higher median PTX3 level in secondary TMA, and this relationship was independent of the hemoglobin and creatinine levels, but was non-independent of platelet and LDH. The difference between median CRP levels did not reach statistical significance in deceased patients compared to those who survived the first month of the TMA episode. This observation conforms published reports in regard to the association of PTX3 to acute disease mortality in multiple conditions including severe sepsis (37, 38), ventilation assisted pneumonia (39) and acute aortic type A dissection (40). Besides, PTX3 was reported to be a long-term prognostic marker of mortality in patients undergoing hemodialysis (41) and of cardiovascular death in patients with renal disease (42), whereas some studies even place PTX3 superior to CRP as a predictor of mortality (39), endothelial dysfunction (43) or indicator of local inflammatory response following vascular injury (44).

Even though both pentraxins have been described to interact with the complement system *in vitro* (1, 2), we only found an association between laboratory signs of complement consumption and elevated PTX3 in the acute phase of TMA. The net result of the PTX3-complement interaction is proposed to be restrain of complement-mediated damage on non-activator surfaces and stimulation of phagocytosis and clearance of cellular debris (1, 2). *In vivo* experimental models of tissue damage however, reported inconclusive data on the overall impact of PTX3 on tissue recovery. In murine models of ischemia-reperfusion injury both endogenous and exogenous PTX3 were described to alleviate leukocyte recruitment following renal ischemia (10), while the lack of PTX3 was associated with a higher degree of apoptosis and C3 deposition in damaged cardiac tissue (11). Nevertheless, others reported that the overexpression or external admission of PTX3 exacerbated the post-ischemic intestinal and remote pulmonary tissue damage (12, 13). In humans PTX3 has been shown to correlate with surrogate markers of disease severity in cardiovascular and renal diseases (35, 45, 46) and molecular characterization of this association suggests that PTX3 is involved in the fine tuning of inflammation with an overall tissue-protective effect (47, 48).

In endothelial damage associated with TMA, although NET formation may promote thrombosis and complement activation (49), as a NET component (29) PTX3 may recruit the complement regulator FH (8) and limit the expansion of tissue damage mediated by the AP. The potential regulatory role of PTX3 on AP activity is suggested by experimental evidence describing FH recruitment by PTX3 (8, 50) to the damaged cell surface, while the presence of anti-FH antibodies or mutations of the complement regulator have been linked to an impaired FH-PTX3 interaction that may aggravate the endothelial damage in aHUS (50). To better understand the potential role of PTX3 elevation in TMA, we measured the changes of AP activity in the presence or absence of external PTX3 using two distinct *in vitro* approaches. First, to determine the AP hemolytic activity, we built up the C3 convertase on sheep erythrocytes under conditions allowing for AP activation only. Second, we assessed C9 generation on the surface of ELISA plates via LPS-induced AP activation, with or without additional PTX3. Based on the gradual decline of the hemolytic activity parallel to the increment of PTX3 concentration in pooled human serum, we conclude that local release of PTX3 may indeed play an important role in the limitation of AP activity. However, based on previously published observations on the interaction of PTX3 with the regulators of complement (8, 9, 50), restrain of the AP activity by PTX3 is most probably due to an indirect effect (e.g., recruitment of complement regulators), which requires cellular attachment of the PRM, rather than direct inhibition of the activation pathway. This hypothesis is also supported by the observed lack of AP restrain, when the activation was induced on the surface of an ELISA plate, however detailed molecular investigation of this phenomenon would be necessary to identify each complement factor involved in the regulatory effect. Nonetheless, the observed restrain of the AP indicates that local release of PTX3 could possibly attenuate complement activity and hence potentially limit the ongoing endothelial damage in TMA patients.

Finally, the lack of correlation between CRP levels and complement consumption could be attributed to the fact, that CRP production is induced in the liver in frames of a systemic inflammatory response that may not closely reflect the degree of local endothelial damage and subsequent complement consumption. However, *in vitro* evidence suggests that through the binding of complement regulators and the restrain of excess terminal pathway activity, CRP as well as PTX3 are able to regulate the AP and CP of complement (1, 15–18).

In conclusion, we report the association of PTX3 elevation with complement overactivation and consumption in TMA. The regulatory role of PTX3 on AP hemolytic activity *in vitro* suggests that PTX3 is an adjunct factor in the prevention of excess endothelial damage in TMA. Our observations are in line with previously published *in vitro* data describing the interaction of PTX3 and individual complement factors, and add to *in vivo* investigations emphasizing the potential tissue-protective role of PTX3. This is the first study where the association of PTX3 and CRP elevation has been investigated in a complement mediated disease *in vivo*, and thus our results provide a missing link between the numerous *in vitro* observations that described the interaction of PTX3 with the complement system under defined experimental conditions. On the other hand, our observations may indicate a potential practical use of PTX3 determination as a biomarker and determinant of complement consumption in the acute phase of TMA. However, apparent limitations of our study are the retrospective enrollment of patients and the rare nature of this disease that together may have caused some of our analyses to be underpowered. The limited number of study subjects and subsequently low case and event numbers in this study precluded multivariate analysis in different etiology based subgroups of TMAs, therefore some of our observation may represent overestimation of true effects due to the lack of adjustment for important clinical and/or laboratory covariates. Therefore, independent confirmation of our observations is necessary before firm conclusions can be reached on the contribution of PTX3 to the pathogenesis of TMA. Nonetheless, the reported association of elevated PTX3 levels and complement consumption may initiate further investigations to understand the exact role of PTX3 in TMA pathogenesis and may aid the better understanding of the heterogeneous clinical course of TMA.

## Author Contributions

ZP and MJ: study concept and design. ET, ZS, NG, BáM, GS, BlM, and DC: experimental procedures. ET, BáM, MR, PF, KK, GR, AS, and ZP: acquisition of data. All authors: analysis and interpretation of data. ET and ZP: critical writing of the manuscript. All authors: critical revision of the manuscript for important intellectual content. ZP and MJ: study supervision. MJ, GR, DC, and ZP: acquisition of funding.

### Conflict of Interest Statement

The authors declare that the research was conducted in the absence of any commercial or financial relationships that could be construed as a potential conflict of interest.
