# Supplementary figures and images for: Elevated Systemic Pentraxin-3 Is Associated With Complement Consumption in the Acute Phase of Thrombotic Microangiopathies
Source: Front Immunol. 2019 Feb 25;10:240. doi: 10.3389/fimmu.2019.00240 (PMC6397851; doi:10.3389/fimmu.2019.00240)

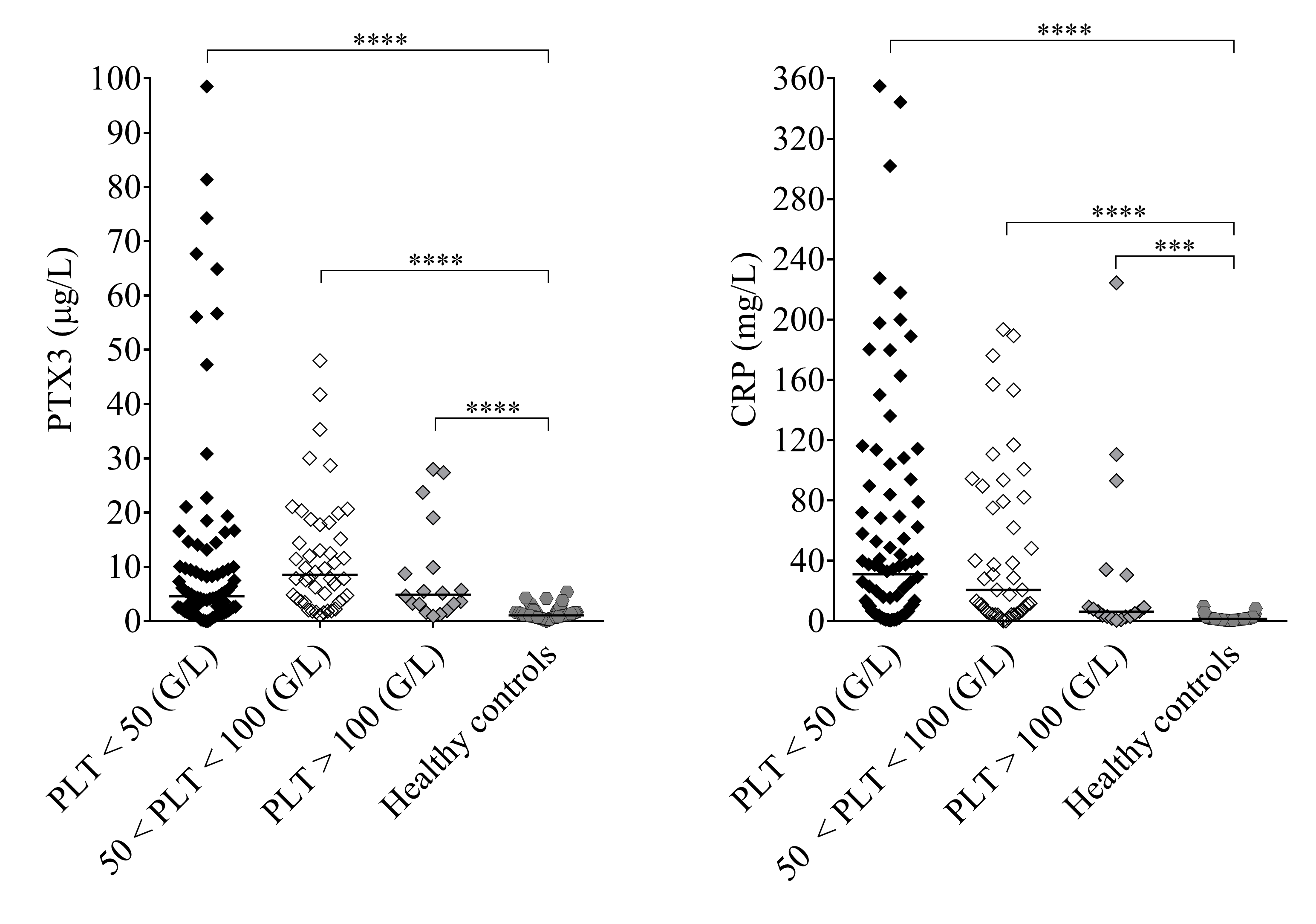

Supplement: Supplementary file 1 [file Image_1.tif]

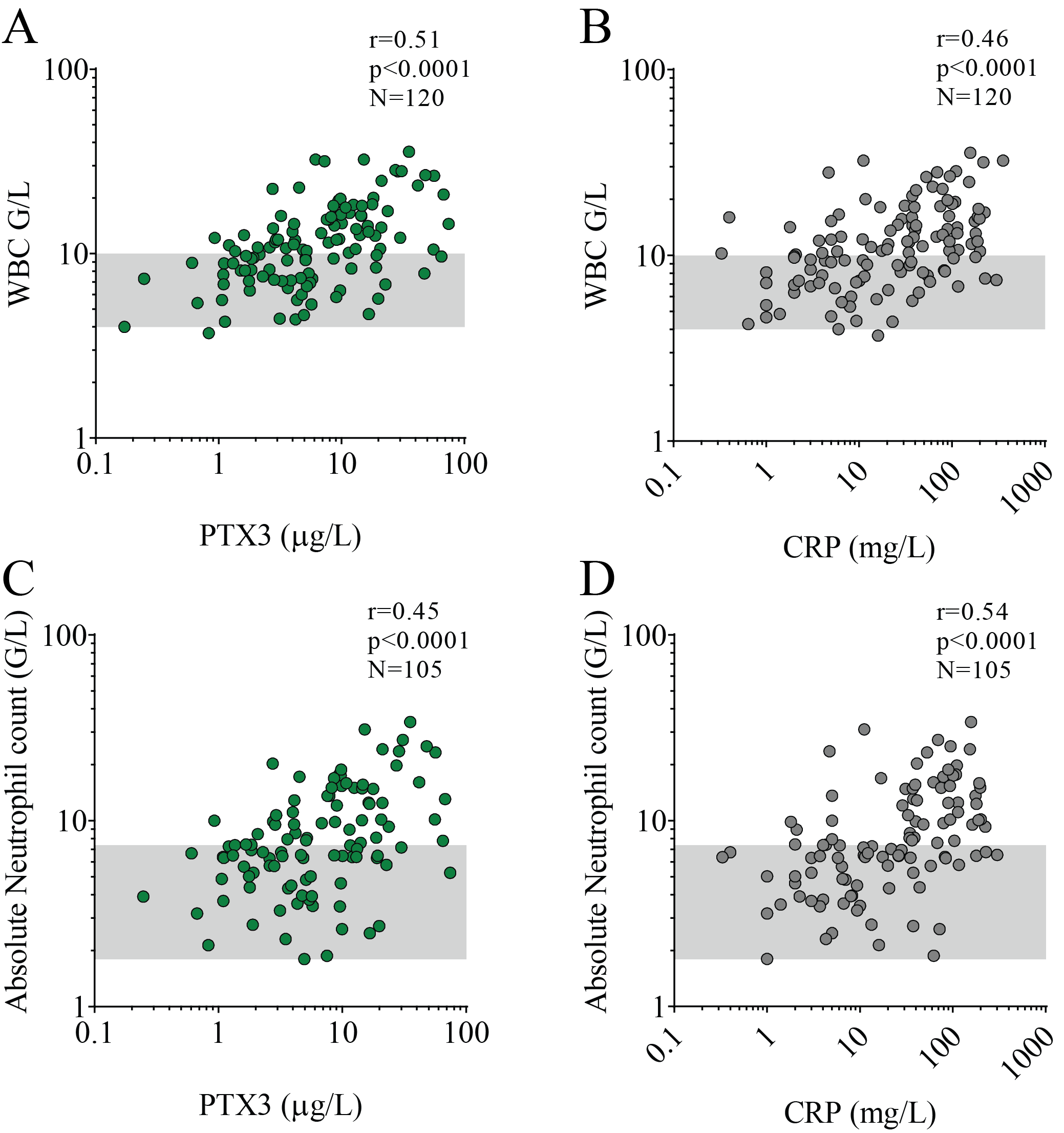

Supplement: Supplementary file 2 [file Image_2.TIF]

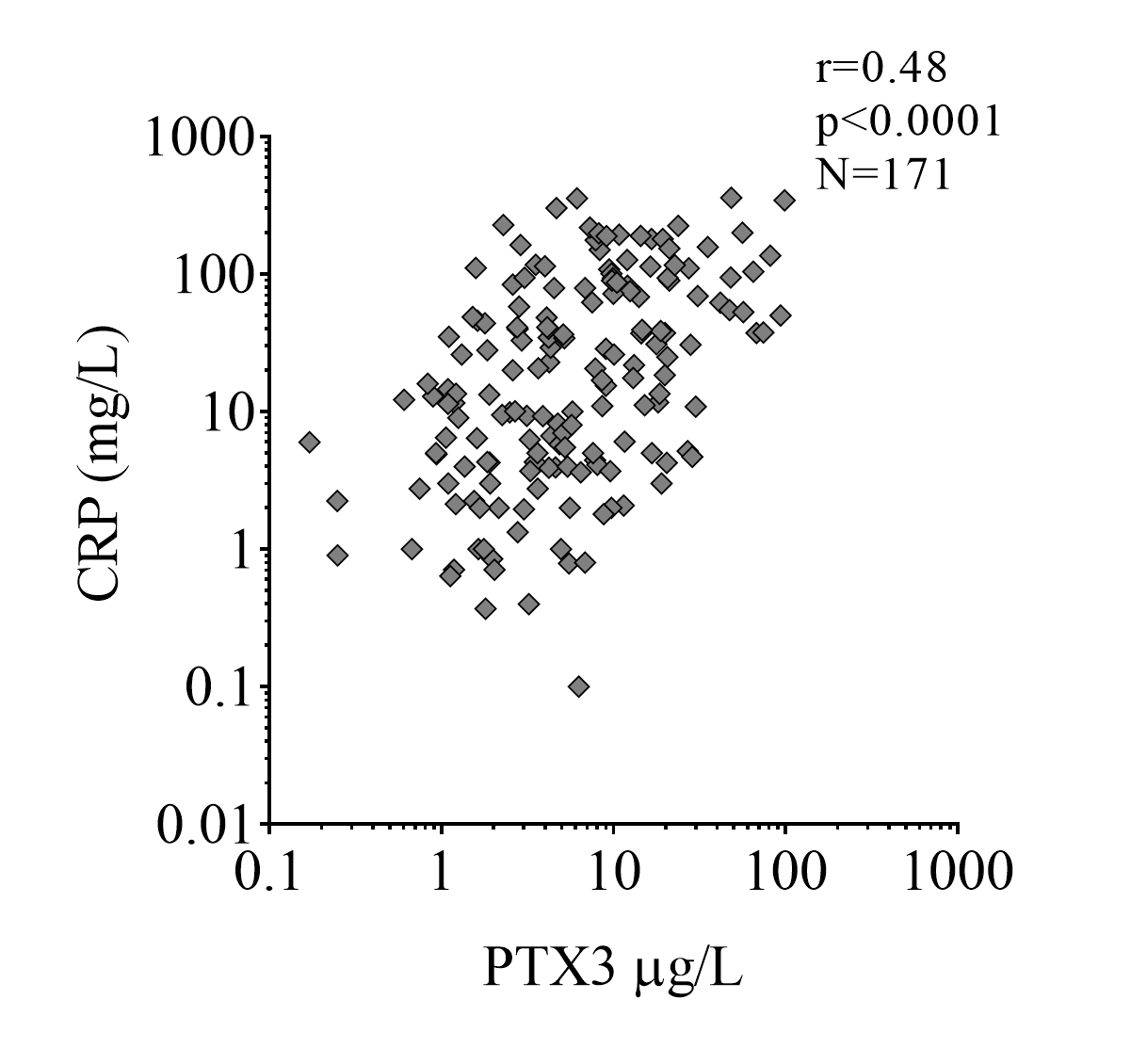

Supplement: Supplementary file 3 [file Image_3.TIF]

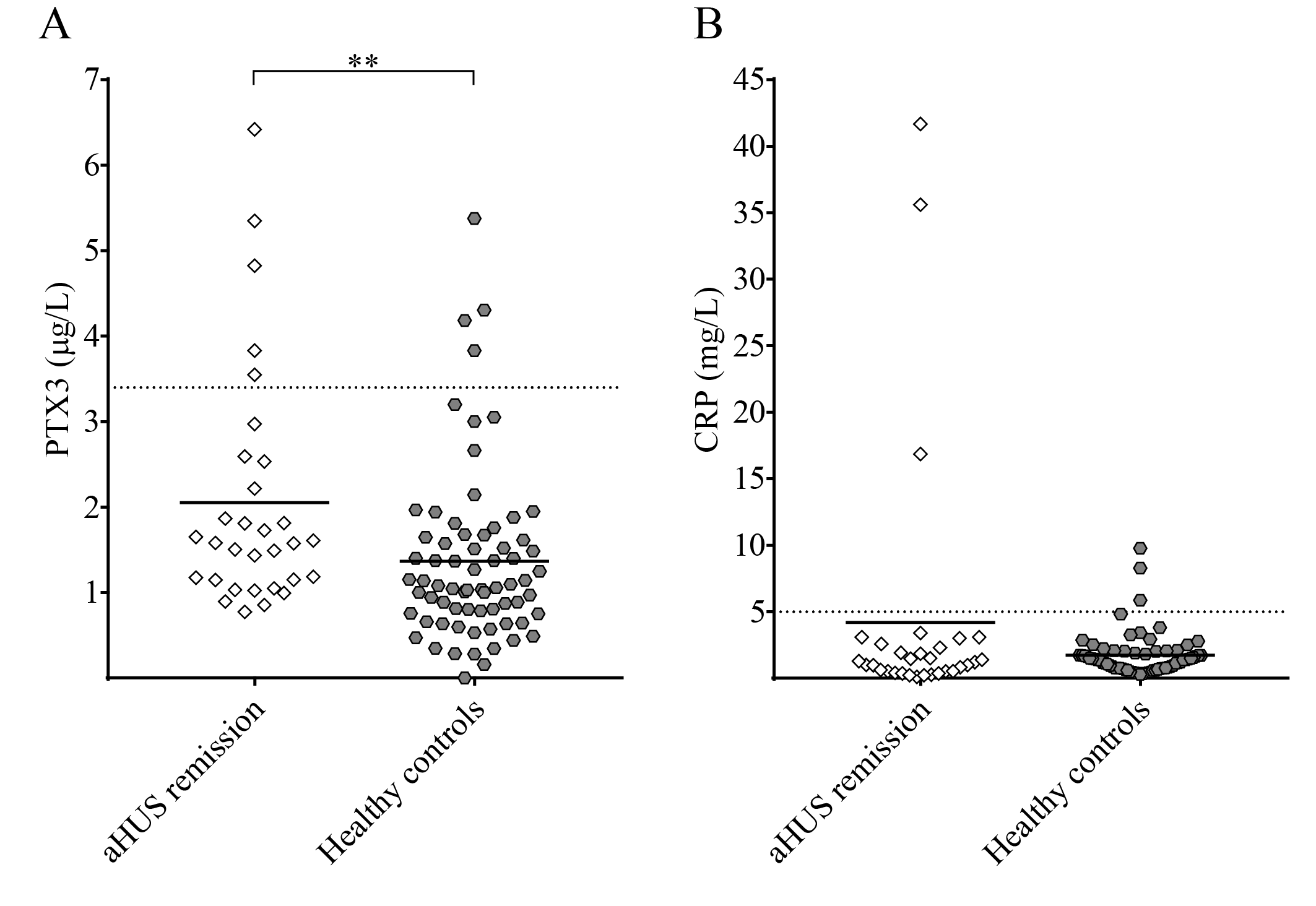

Supplement: Supplementary file 4 [file Image_4.TIF]

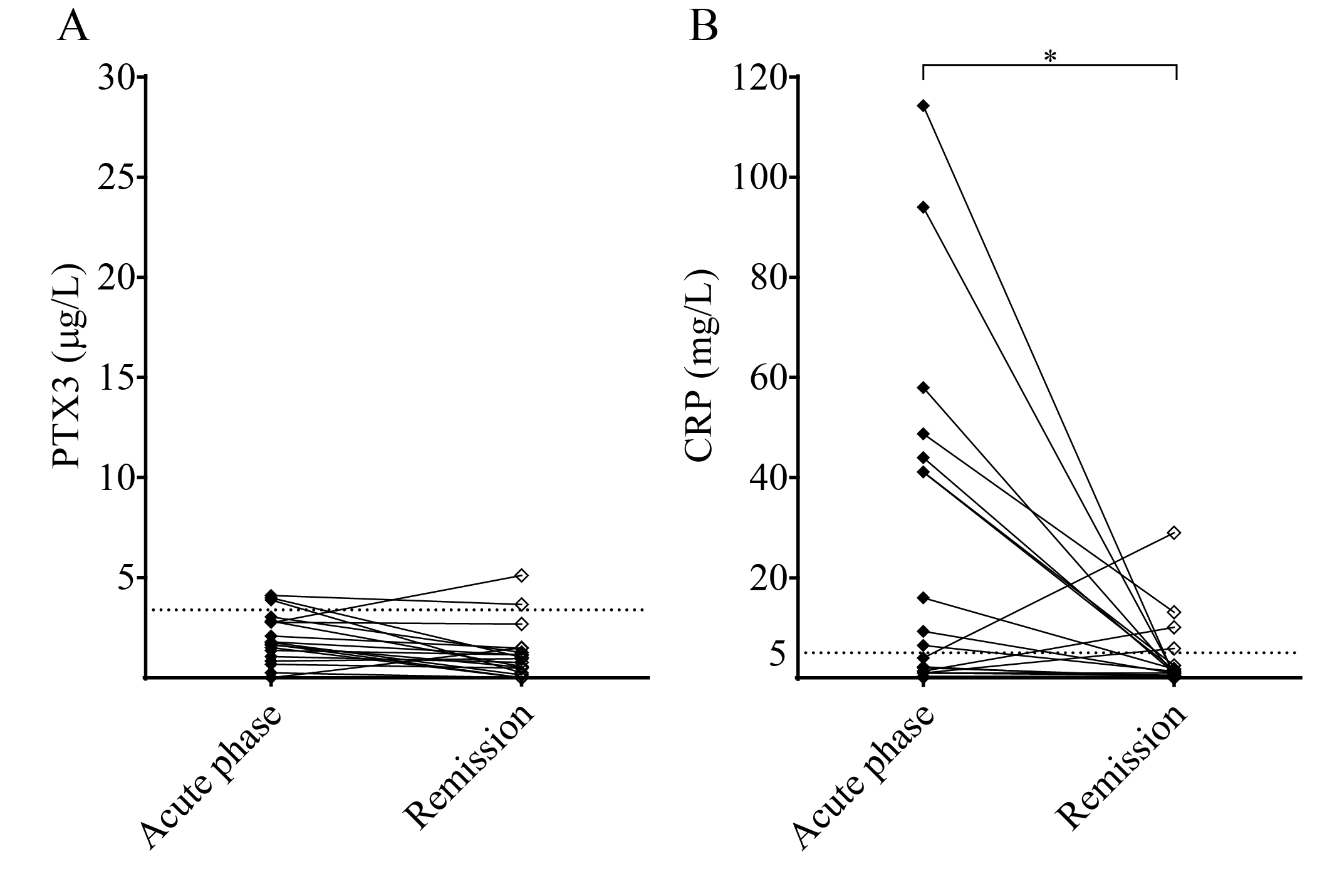

Supplement: Supplementary file 5 [file Image_5.TIF]

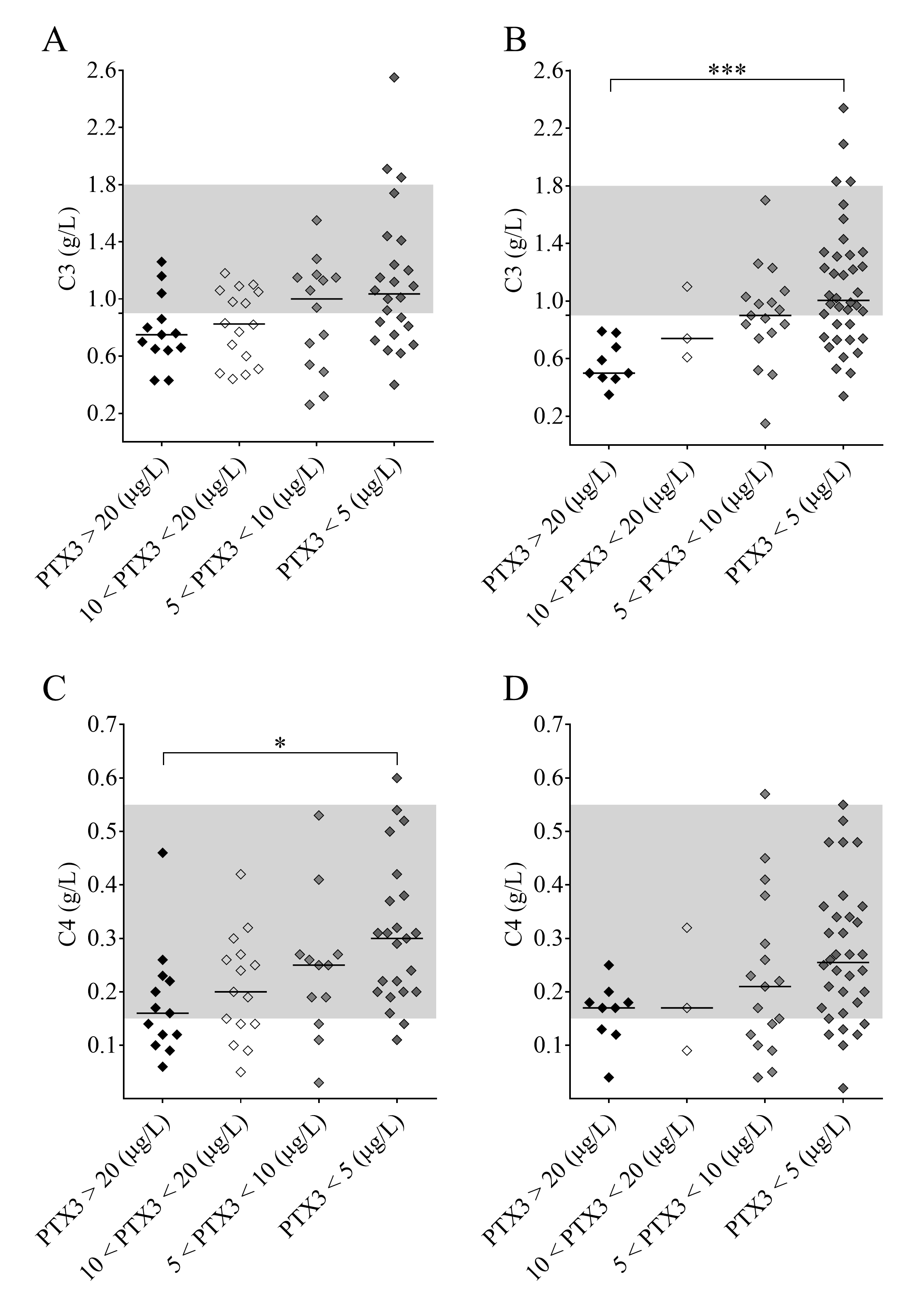

Supplement: Supplementary file 6 [file Image_6.TIF]
