# Supplementary material for: Elevated Systemic Pentraxin-3 Is Associated With Complement Consumption in the Acute Phase of Thrombotic Microangiopathies
Source: Front Immunol. 2019 Feb 25;10:240. doi: 10.3389/fimmu.2019.00240 (PMC6397851; doi:10.3389/fimmu.2019.00240)
Supplement: Supplementary file 7 [file Data_Sheet_1.pdf]

Supplementary Material

Elevated systemic pentraxin-3 is associated with complement consumption in the acute phase of thrombotic microangiopathies

Methods

Patient selection and sample collection

Acute phase thrombotic microangiopathy (TMA) patients enrolled in this study were selected from subjects whose blood samples were sent to our laboratory for differential diagnostic evaluation between November 2007 and October 2017. Patient stratification based on TMA disease etiology was conducted as follows: (1) Shiga-like toxin associated hemolytic uremic syndrome (STEC-HUS): acute gastroenteritis and signs of acute kidney injury with proof of Shiga like-toxin producing *Escherichia coli* infection based on either the demonstration of Shiga-like toxins 1 or 2 by PCR from stool, or identification of pathogenic STEC from stool culture and verification of toxin production by PCR or immunoassay, (2) thrombotic thrombocytopenic purpura (TTP): deficiency (activity below 10%) of a disintegrin and metalloproteinase with a thrombospondin type 1 motif member 13 (ADAMTS13) with the presence of ADAMTS13 inhibitors, (3) atypical hemolytic uremic syndrome (aHUS): HUS with presence of anti-factor H autoantibodies, or HUS with identified pathogenic or likely pathogenic variations in the complement genes (*CFH, CFHR5, CFI, CD46, C3, CFB*) or the genes encoding thrombomodulin (*THBD*) or diacylglycerol kinase epsilon (*DGKE*), or HUS cases without identified likely pathogenic rare variations in the complement genes, or the genes encoding *THBD* or *DGKE*, (4) secondary TMA: evidence of coexisting disease including malignancy, autoimmune disease, sepsis, solid organ transplantation, open heart surgery or malignant hypertension. Genetic analysis was performed by direct DNA sequencing of polymerase chain reaction products amplified from total genomic DNA. Pathogenic and likely pathogenic mutations were defined based on published literature data and included mutations either described previously in aHUS patients or expected to cause aHUS based on functional analysis reported in literature.

Acute kidney injury was defined as documented oligo- or anuria or creatinine and carbamide levels above the upper limit of the laboratory normal range. Data on the clinical course, blood count and chemistry were collected from the medical charts. Patients were followed-up after hospital discharge and outcome, including mortality, was registered. Follow-up samples in remission were available for aHUS patients (N=31, 15-month median time interval between sampling) and TTP patients (N=19, 8-month median time interval between sampling), each collected at least 3 months following the last acute episode of the patients, respectively. Whole blood samples were immediately separated after collection (to yield serum, EDTA-anticoagulated plasma, and sodium-citrate-anticoagulated plasma) and stored in aliquots at −70°C until further analysis.

Statistical analysis

Since continuous variables of this study failed the Shapiro-Wilk's normality test, non-parametric tests were carried out for group comparisons with two-tailed p-values calculated, and the significance level set at 0.05. For descriptive purposes, continuous variables are shown as median with interquartile range, while categorical variables are indicated with numbers (expressed in percentage). Multiple linear regression analysis (on log-transformed variables) was used to explore the relationship between covariates of pentraxin 3 and CRP levels. Logistic

regression analysis with one-by-one adjustment for covariates was used to test dichotomized pentraxin levels as predictor of mortality.

Supplementary Table 1 Characteristic laboratory parameters of TMA with distinct etiology

Laboratory parameters indicative of disease activity (hemolysis, thrombocytopenia, and kidney impairment) are shown in each TMA group. Data are expressed as median and interquartile range. Please note that blood count and chemistry data were not accessible by all the enrolled patients, therefore the analysis might include less than the total number of patients (N=171) in this study. (BUN= blood urea nitrogen, Crea= creatinine, Hb= hemoglobin, aHUS= atypical hemolytic uremic syndrome, LDH= lactate dehydrogenase, PLT= platelet count, RBC= red blood cell count, STEC-HUS= Shiga-like toxin associated HUS, TMA=thrombotic microangiopathy, TTP= thrombotic thrombocytopenic purpura, WBC= White blood cell count, ANC= Absolute neutrophil count)

Laboratory parameters	aHUS	N	STEC-HUS	N	Secondary TMA	N	TTP	N
**RBC (T/L)**	2.8 (2.5-3.3)	17	3.3 (2.8-3.7)	31	2.9 (2.6-3.3)	49	2.7 (2.1-3.2)	24
**Hb (g/L)**	80 (67-95)	30	87 (76-101)	33	88 (79-95)	53	80 (67-99)	29
**PLT (G/L)**	53 (29-86)	32	51 (33-88)	33	50 (34-76)	54	16 (11-27)	29
**LDH (U/L)**	2777 (1806-4027)	24	3280 (2374-5157)	30	1005 (545-1634)	51	1444 (819-2370)	29
**Crea (µmol/L)**	269 (113-535)	37	292 (166-489)	32	188 (89-302)	58	79 (68-100)	29
**BUN (mmol/L)**	20.3 (13.0-25.7)	24	19.0 (14.2-40.1)	32	17.9 (11.7-28.0)	46	6.5 (5.0-9.0)	21
**WBC (G/L)**	10.1 (6.3-14.1)	19	12.4 (9.4-15.7)	32	10.9 (6.0-18.1)	52	8.85 (7.1-10.3)	28
**ANC (G/L)**	6.8 (3.6-12.2)	14	6.5 (5.3-9.7)	32	9.9 (4.7-15.9)	44	6.5 (4.6-7.9)	24

Supplementary Table 2

Complement factor levels and activity parameters of each TMA subgroup displayed corresponding to Figure 5. Data are grouped based on the patients' PTX3 **(A)** and CRP **(B)** levels, and are shown as median with interquartile range (AP= alternative pathway, aHUS= atypical hemolytic uremic syndrome, CP= classical pathway, CRP= C-reactive protein, PTX3= pentraxin-3, STEC-HUS= Shiga-like toxin associated HUS, TMA= thrombotic microangiopathy, TTP= thrombotic thrombocytopenic purpura)

A

**aHUS**	**PTX3 > 20 (μg/L)**	**10 < PTX3 < 20 (μg/L)**	**5 < PTX3 < 10 (μg/L)**	**PTX3 < 5 (μg/L)**
**C3**	0.76 (0.68-0.76)	0.79 (0.68-1.10)	0.69 (0.52-1.00)	0.89 (0.68-1.01)
**C4**	0.10 (0.06-1.20)	0.25 (0.19-0.27)	0.25 (0.19-0.26)	0.29 (0.19-0.38)
**Factor H**	421 (282-439)	64 (30-72)	250 (115-599)	379 (183-548)
**AP activity**	59 (56-85)	87 (7-94)	56 (11-98)	96 (64-105)
**CP activity**	47 (37-58)	64 (30-72)	54 (42-74)	57 (50-65)
**N**	5	7	9	23
**STEC-HUS**	**PTX3 > 20 (μg/L)**	**10 < PTX3 < 20 (μg/L)**	**5 < PTX3 < 10 (μg/L)**	**PTX3 < 5 (μg/L)**
**C3**	0.66 (0.50-1.04)	0.98 (0.66-1.10)	1.26 (1.07-1.44)	1.01 (0.83-1.26)
**C4**	0.12 (0.10-0.12)	0.24 (0.12-0.37)	0.19 (0.10-0.41)	0.24 (0.18-0.38)
**Factor H**	272 (160-494)	393 (303-513)	342 (237-450)	387 (254-453)
**AP activity**	49 (2-81)	77 (69-101)	98 (88-109)	91 (67-99)
**CP activity**	39 (23-46)	54 (46-76)	65 (59-79)	60 (44-67)
**N**	7	10	7	10
**secondary TMA**	**PTX3 > 20 (μg/L)**	**10 < PTX3 < 20 (μg/L)**	**5 < PTX3 < 10 (μg/L)**	**PTX3 < 5 (μg/L)**
**C3**	0.59 (0.47-0.77)	0.74 (0.53-0.98)	0.88 (0.73-1.03)	0.9 (0.69-1.03)
**C4**	0.18 (0.15-0.23)	0.14 (0.11-0.25)	0.21 (0.11-0.26)	0.26 (0.13-0.33)
**Factor H**	289 (184-437)	280 (222-387)	350 (200-479)	409 (313-566)
**AP activity**	34 (18-55)	72 (54-108)	74 (43-98)	73 (56-95)
**CP activity**	42 (32-56)	53 (39-66)	60 (36-78)	59 (45-70)
**N**	13	9	19	22
**TTP**	**PTX3 > 20 (μg/L)**	**10 < PTX3 < 20 (μg/L)**	**5 < PTX3 < 10 (μg/L)**	**PTX3 < 5 (μg/L)**
**C3**	1.26	NA	1.15	1.32 (1.11-1.77)
**C4**	0.46	NA	0.53	0.27 (0.19-0.44)
**Factor H**	1182	NA	473	504 (384-755)
**AP activity**	115	NA	115	101 (93-111)

**CP activity**	91	NA	67	76 (59-87)
**N**	1	0	1	28

B

**aHUS**	**CRP > 20 (mg/L)**	**10 < CRP < 20 (mg/L)**	**5 < CRP < 10 (mg/L)**	**CRP < 5 (mg/L)**
**C3**	0.89 (0.77-1.09)	0.83 (0.70-1.09)	0.87 (0.68-0.94)	0.71 (0.64-1.07)
**C4**	0.25 (0.18-0.29)	0.27 (0.23-0.43)	0.27 (0.12-0.35)	0.23 (0.18-0.34)
**Factor H**	443 (275-679)	245 (60-443)	435 (250-513)	230 (109-605)
**AP activity**	94 (81-100)	64 (27-100)	96 (62-105)	62 (51-96)
**CP activity**	59 (50-69)	57 (25-62)	54 (49-65)	52 (44-66)
**N**	12	5	7	20
**STEC-HUS**	**CRP > 20 (mg/L)**	**10 < CRP < 20 (mg/L)**	**5 < CRP < 10 (mg/L)**	**CRP < 5 (mg/L)**
**C3**	0.98 (0.66-1.14)	0.96 (0.78-1.06)	0.86 (0.82-1.38)	1.17 (1.03-1.27)
**C4**	0.18 (0.11-0.32)	0.23 (0.15-0.35)	0.23 (0.21-0.37)	0.17 (0.09-0.39)
**Factor H**	355 (233-447)	353 (270-511)	328 (212-420)	429 (263-518)
**AP activity**	74 (54-96)	86 (67-103)	83 (35-105)	96 (88-106)
**CP activity**	46 (35-70)	53 (42-53)	63 (60-65)	62 (50-75)
**N**	18	4	4	8
**secondary TMA**	**CRP > 20 (mg/L)**	**10 < CRP < 20 (mg/L)**	**5 < CRP < 10 (mg/L)**	**CRP < 5 (mg/L)**
**C3**	0.75 (0.52-0.93)	0.72 (0.56-0.95)	1.04 (0.6-1.56)	0.97 (0.74-1.2)
**C4**	0.18 (0.12-0.26)	0.23 (0.13-0.27)	0.34 (0.15-0.45)	0.24 (0.10-0.34)
**Factor H**	319 (210-441)	296 (267-393)	445 (342-572)	467 (298-652)
**AP activity**	64 (33-86)	64 (43-85)	90 (43-113)	95 (48-107)
**CP activity**	50 (37-62)	58 (43-72)	69 (54-90)	61 (30-92)
**N**	41	8	4	10
**TTP**	**CRP > 20 (mg/L)**	**10 < CRP < 20 (mg/L)**	**5 < CRP < 10 (mg/L)**	**CRP < 5 (mg/L)**
**C3**	1.31 (1.22-1.83)	1.76	1.06	1.26 (0.93-1.64)
**C4**	0.4 (0.23-0.52)	0.37	0.2	0.27 (0.14-0.4)
**Factor H**	739 (452-847)	367	474	504 (406-767)
**AP activity**	111 (96-115)	102	94	103 (90-110)
**CP activity**	86 (70-88)	57	70	76 (54-98)
**N**	11	2	3	14

Supplementary Figure 1. Association of the systemic pentraxin levels with the platelet count of TMA patients

PTX3 **(A)** and CRP **(B)** levels of acute phase-TMA patients are shown, subdivided based on the platelet count at disease onset. Data are expressed as mean of technical duplicates, the horizontal line indicates the median of each group. Statistical analysis was performed with the Kruskal-Wallis test corrected for multiple comparisons using the Dunn's post hoc test. Statistical significance is indicated by asterisks (***p<0.001, ****p<0.0001). (PTX3= pentraxin-3, TMA=thrombotic microangiopathy)

Supplementary Figure 2. Correlation of pentraxin levels to inflammatory parameters.

Correlation of the PTX3 (green circles) and the CRP (grey circles) levels to the WBC **(A-B)** and absolute neutrophil count **(C-D)** of the patients. Laboratory normal ranges are shown with grey shading. To avoid false negative results, patients with a WBC below 4 G/L (N=10) and those with an absolute neutrophil count below 1.8 G/L (N=5) were excluded from this analysis. Statistical analysis was performed using the Spearman correlation test. Respective r and p values are indicated in the upper right corner of each graph. Please note that blood count and chemistry data were not available by all the enrolled patients, therefore the correlation analyses might include less than the total number of patients (N=171) in this study. Patients with an undetectable PTX3 or CRP level (N=3) are not shown on the log-log scale, however their data were included in the statistical analysis. (CRP= C-reactive protein, PTX3= pentraxin-3, WBC= white blood cell count)

Supplementary Figure 3. Correlation of the systemic pentraxin levels to each other.

Correlation of the PTX3 levels to the CRP levels is plotted on a log-log scale. Statistical analysis was performed using the Spearman correlation test. The r and p values are indicated in the upper right corner of the graph. Patients with an undetectable PTX3 or CRP level (N=3) are not shown on the log-log scale, however their data were included in the statistical analysis. (CRP= C-reactive protein, PTX3= pentraxin-3)

Supplementary Figure 4. PTX3 and CRP levels in aHUS remission compared to healthy controls.
PTX3 **(A)** and CRP **(B)** levels of aHUS patients in remission (empty squares) compared to healthy controls (grey hexagons). Data represent mean of technical duplicates, the horizontal line indicates the median of each group, while an intermittent line shows the calculated cutoff of each pentraxin, respectively. Statistical analysis was performed with the Mann-Whitney test. Statistical significance is indicated by asterisks (**p<0.01). (CRP= C-reactive protein, PTX3= pentraxin-3, aHUS= atypical hemolytic uremic syndrome)

Supplementary Figure 5. PTX3 and CRP levels in TTP acute phase and remission.
PTX3 **(A)** and CRP **(B)** levels of TTP patients are displayed in the acute phase (black squares) and in remission (empty squares) with a continuous line connecting the respective sample pairs. Data represent mean of technical duplicates, statistical analysis was performed with the Wilcoxon-signed rank test. Statistical significance is indicated by asterisks (*p<0.05). (CRP= C-reactive protein, PTX3= pentraxin-3, TTP= thrombotic thrombocytopenic purpura)

Supplementary Figure 6. Association of the systemic PTX3 levels to C3 and C4 in TMA patients stratified according to LDH.

Acute phase TMA patients C3 **(A-B)** and C4 **(C-D)** levels are displayed, subdivided based on their systemic PTX-3 and LDH levels. Panels **A** and **C** show the measured complement parameters of those patients who had an LDH>1819 U/L (The calculated median LDH level in TMA patients was 1819 U/L), whereas panels **B** and **D** include those with an LDH<1819 U/L. Data are expressed as mean of technical duplicates, the horizontal lines show the median of each group and the laboratory normal range is indicated with grey shading. Statistical analysis was performed with the Kruskal-Wallis test corrected for multiple comparisons using the Dunn's post hoc test. Statistical significance is indicated by asterisks (*p<0.05, ***p<0.001).
